# The Central New York Medical Association

**Published:** 1891-07

**Authors:** 


The Central New York Medical Association held its twenty-fourth annual session in Buffalo, June 2, 1891. The meeting was largely attended, and under the efficient presidency of Dr. Nathan Jacobson, of Syracuse, a comprehensive and interesting programme was despatched. This was its first meeting in Buffalo, and the new constituent of the association, Erie county, gave it a hearty welcome. The following officers were elected for the ensuing year: President, Alvin A. Hubbell, M. D., Buffalo; First Vice-President

Lewis A. Weigel, M. D., Rochester ; Second Vice-President, Henry L. Elsner, M. D., Syracuse ; the Secretary, Edward B. Angell, M. D., Rochester, and Treasurer, Alfred Mercer, M. D., Syracuse, hold over.

Hereafter only one meeting will be held each year, and that, on the first Tuesday of June. The meetings will rotate between Buffalo, Syracuse and Rochester. The next one will be held in Syracuse. The association now comprises sixteen counties of central and western New York.

